# Inhibition of PLK1 Destabilizes EGFR and Sensitizes EGFR-Mutated Lung Cancer Cells to Small Molecule Inhibitor Osimertinib

**DOI:** 10.3390/cancers15092589

**Published:** 2023-05-02

**Authors:** Carolien Eggermont, Gustavo J. Gutierrez, Jacques De Grève, Philippe Giron

**Affiliations:** 1 Laboratory of Medical and Molecular Oncology, Oncology Research Center, Faculty of Medicine and Pharmacy, Vrije Universiteit Brussel, Laarbeeklaan 103, 1090 Brussels, Belgium; 2 Laboratory of Pathophysiological Cell Signaling, Department of Biology, Faculty of Science and Bioengineering Sciences, Vrije Universiteit Brussel, Pleinlaan 2, 1050 Brussels, Belgium; 3 Centre for Medical Genetics, Research Group Reproduction and Genetics, Clinical Sciences, UZ Brussel, Vrije Universiteit Brussel, Laarbeeklaan 101, 1090 Brussels, Belgium

**Keywords:** non-small cell lung cancer, PLK1, EGFR, c-Cbl, Osimertinib, Volasertib

## Abstract

**Simple Summary:**

Despite the clinical use of epidermal growth factor receptor (EGFR) inhibitors for non-small cell lung cancer (NSCLC) patients, this disease remains incurable due to the development of resistance mechanisms to treatment. We demonstrate that the inhibition of polo-like kinase 1 (PLK1), known as a master cell cycle regulator, decreases EGFR protein levels in NSCLC cell lines. Better inhibition of EGFR-mutant lung cancer cells was observed with the combination of EGFR and PLK1 inhibitors compared to EGFR inhibition alone. This might therefore be a more potent therapy option to improve the outcomes of patients with EGFR-mutated NSCLC.

**Abstract:**

Tyrosine kinase inhibitors (TKI) targeting the epidermal growth factor receptor (EGFR) have significantly prolonged survival in EGFR-mutant non-small cell lung cancer patients. However, the development of resistance mechanisms prohibits the curative potential of EGFR TKIs. Combination therapies emerge as a valuable approach to preventing or delaying disease progression. Here, we investigated the combined inhibition of polo-like kinase 1 (PLK1) and EGFR in TKI-sensitive EGFR-mutant NSCLC cells. The pharmacological inhibition of PLK1 destabilized EGFR levels and sensitized NSCLC cells to Osimertinib through induction of apoptosis. In addition, we found that c-Cbl, a ubiquitin ligase of EGFR, is a direct phosphorylation target of PLK1 and PLK1 impacts the stability of c-Cbl in a kinase-dependent manner. In conclusion, we describe a novel interaction between mutant EGFR and PLK1 that may be exploited in the clinic. Co-targeting PLK1 and EGFR may improve and prolong the clinical response to EGFR TKI in patients with an EGFR-mutated NSCLC.

## 1. Introduction

The epidermal growth factor receptor (EGFR) signaling pathway regulates several critical cellular mechanisms, including cell survival and proliferation. In about 15% of Caucasian non-small cell lung cancer (NSCLC) patients, the mutational activation of EGFR is a known oncogenic driver [1,2,3]. The prognosis of patients with EGFR-mutant NSCLC has dramatically improved since EGFR tyrosine kinase inhibitors (TKIs) were introduced in the clinical setting as first-line therapy, and even more so with the potent third-generation drug Osimertinib [4].

Despite the clinical success of EGFR TKIs, tolerance and resistance mechanisms invariably lead to disease progression in treated patients [5,6,7]. Acquired resistance due to secondary EGFR mutations, MET amplification, or small-cell lung cancer transformation are well known, but the array of resistance mechanisms is much more diverse and many mechanisms remain unknown [5], highlighting the need for novel upfront combination therapies to avoid the emergence of acquired drug resistance. 

Polo-like kinase 1 (PLK1) has been extensively studied as a target of interest for anti-cancer therapy, considering its role as a master cell cycle regulator. PLK1 is a serine/threonine kinase that regulates several cellular processes such as entry into mitosis, centrosome maturation, spindle assembly, chromosome segregation, and cytokinesis [8]. PLK1 is overexpressed in multiple cancers, including NSCLC, and its overexpression is directly associated with poor survival outcomes [9,10]. In the context of wild-type EGFR NSCLC cells, the synergism between EGFR inhibition (using gefitinib) and PLK1 inhibitors was shown in the context of paclitaxel-resistance, and a cetuximab-conjugated nanoparticle delivering PLK1 siRNA was also shown to sensitize NSCLC cells to radiotherapy [11,12]. Moreover, we have previously shown that EGFR-mutant NSCLC cells are sensitive to PLK1 inhibitors, which cause G2/M cell cycle arrest and apoptosis [13]. In addition, the dual targeting of EGFR and PLK1 has demonstrated a benefit in NSCLC cells with acquired resistance to EGFR TKI [14,15]. Therefore, PLK1 appears to be an attractive potential co-target for initial EGFR TKI-based therapies in NSCLC. 

In this study, we aimed to better understand the molecular and cellular effects of PLK1 inhibition on the EGFR pathway in NSCLC cells. We show that PLK1 stabilizes (mutant) EGFR and that co-targeting of PLK1 and EGFR leads to an increased therapeutic effect due to the induction of apoptosis. Moreover, we found that the E3 ubiquitin ligase of EGFR, namely c-Cbl, is a direct phosphorylation target of PLK1 and the kinase active PLK1 influences c-Cbl stability. The inhibition of c-Cbl by PLK1 leads to the destabilization of EGFR, albeit by a further to-be-elucidated mechanism. PLK1 can be considered for combination therapy with EGFR TKI to improve the initial treatment efficacy in EGFR-mutant NSCLC.

## 2. Results

### 2.1. PLK1 Inhibition Decreases EGFR Protein Levels in NSCLC Cells

In previous work, we showed a synthetic lethal combination of PLK1 inhibition and TRAIL in EGFR-mutant NSCLC cell lines [13]; however, the impact of PLK1 targeting on the EGFR signaling pathway remained unexplored. In the current study, we aimed to investigate the potential role of PLK1 inhibition in EGFR-mutant NSCLC. We used two EGFR-mutant NSCLC cell lines, PC9 and H1975, which harbor the exon 19 deletion (Δ746-750) and the L858R and T790M mutations in EGFR, respectively. We tested two independent ATP-competitive PLK1 inhibitors, RO3280, under pre-clinical evaluation, and Volasertib, a widely used PLK1 inhibitor in advanced clinical development with manageable adverse events. 

**Figure 1:**
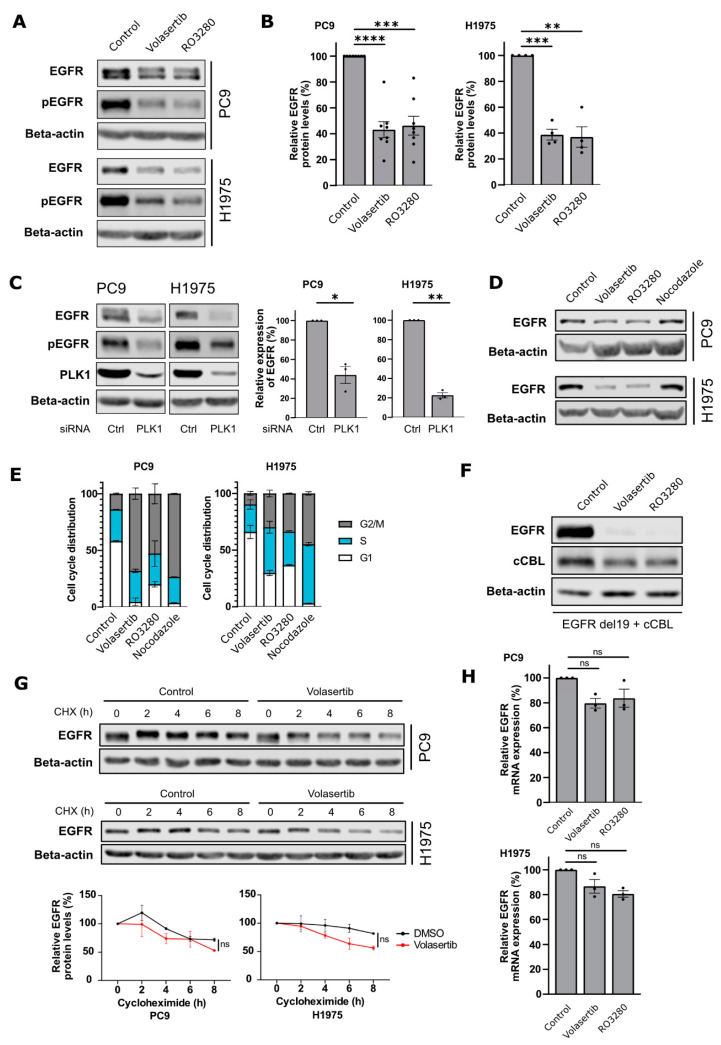
PLK1 inhibition decreases mutant EGFR protein levels, independent of cell cycle arrest. (**A**,**B**) PC9 and H1975 cells were treated with Volasertib or RO3280 at 80 nM for 24 h and (**A**) were analysed using Western blot for the indicated antibodies. (**B**) Relative quantifications of EGFR levels are shown as mean ± SEM (dots represent independent experiments; PC9: *n* = 9 and H1975: *n* = 5). (**C**) PC9 and H1975 cells were transfected with control (ctrl) or PLK1 siRNA and lysed after 48 h. Lysates were analysed using Western blot for the indicated antibodies. (**D**,**E**) PC9 and H1975 cells were treated with Volasertib (80 nM), RO3280 (80 nM), or Nocodazole (200 ng/mL) for 24 h. (**D**) Cell lysates were analyzed with Western blot and (**E**) the relative cell cycle distribution as determined by 7-AAD staining in 2 independent experiments is shown. (**F**) HEK293T cells were transfected with EGFR del746-750 and c-Cbl and treated overnight with Volasertib (80 nM) or RO3280 (80 nM). After a total incubation time of 24 h, cells were lysed and analyzed using Western blotting. (**G**) PC9 and H1975 cells were pre-treated with Volasertib (80 nM) or control for 11 h before the addition of cycloheximide (40 µg/mL) for multiple time points. EGFR protein levels were quantified and normalized to beta-actin levels. Graphs show the mean ± SEM of two independent experiments. (**H**) PC9 and H1975 cells were treated with 80 nM Volasertib or RO3280 and lysed after 24 h incubation. Relative mRNA levels of EGFR were determined using qRT-PCR. Data are presented as mean ± SEM from 3 independent experiments. Dots represent individual experiments. (ns= non-significant; * *p* < 0.05; ** *p* < 0.01; *** *p* < 0.001; **** *p* < 0.0001).

In both of our NSCLC cell line models, PC9 and H1975, either RO3280 or Volasertib treatment resulted in decreased levels of total EGFR, as observed by Western blotting (Figure 1A,B). PLK1 protein levels increased upon PLK1 inhibition both with RO3280 and Volasertib (Supplemental Figure S1A). The decrease in EGFR correlated with the concentration and duration of the PLK1 inhibitor treatment (Supplemental Figure S1B,C). Furthermore, the decline in the total levels of EGFR was associated with a decrease in its activation (Figure 1A). SiRNA knockdown of PLK1 also significantly decreased EGFR and phospho-EGFR levels, confirming a PLK1-specific effect (Figure 1C).

Next, we investigated the mechanism underlying the reduction in EGFR protein levels upon PLK1 inhibition. PLK1 inhibition has been described in the literature to cause cell cycle arrest. Our experiments confirmed that RO3280 as well as Volasertib treatment led to a G2/M arrest (Figure 1D,E and Supplemental Figure S2). To distinguish whether the reduction in EGFR levels was specific to PLK1 inhibition or rather a consequence of the G2/M arrest, we compared the EGFR protein levels in cells treated with RO3280 and Volasertib versus cells treated with Nocodazole, a small molecule compound leading to a similar cell cycle arrest due to interference with the polymerization of microtubules. In both PC9 and H1975 cells, despite achieving a similar cell cycle arrest in all treatment conditions, we only observed decreased EGFR protein levels upon treatment with PLK1 inhibitors but not upon treatment with Nocodazole (Figure 1D,E and Supplemental Figure S2). These data indicate that the reduction in EGFR protein levels is a direct effect of PLK1 inhibition, and independent of the G2/M arrest induced by PLK1 inhibition.

Next, we also found that EGFR-Δ746-750 (exon 19 deletion) protein levels, when expressed in HEK293T cells under a constitutive CMV promoter, were decreased upon PLK1 inhibition, supporting a post-translational mechanism (Figure 1F). In the same experiment, we also expressed c-Cbl, a well-known E3 ubiquitin ligase implicated in EGFR lysosomal degradation, and observed that c-Cbl protein levels were reduced upon PLK1 inhibition (Figure 1F). To further evaluate the stability of EGFR upon PLK1 inhibition, we conducted a cycloheximide experiment with or without Volasertib in PC9 and H1975 cells. We observed that PLK1 inhibition decreased the half-life of EGFR; however, this trend was not statistically significant upon quantification (Figure 1G). 

Moreover, we performed quantitative real-time PCR analyses to assess the potential transcriptional effects of PLK1 inhibition. We found no statistically significant differences in EGFR mRNA transcript levels upon Volasertib and RO3280 treatments (Figure 1H). 

Overall, our data establish that inhibition of PLK1 negatively impacts the protein levels of mutant EGFR. 

### 2.2. PLK1 Interacts with and Phosphorylates c-Cbl

**Figure 2:**
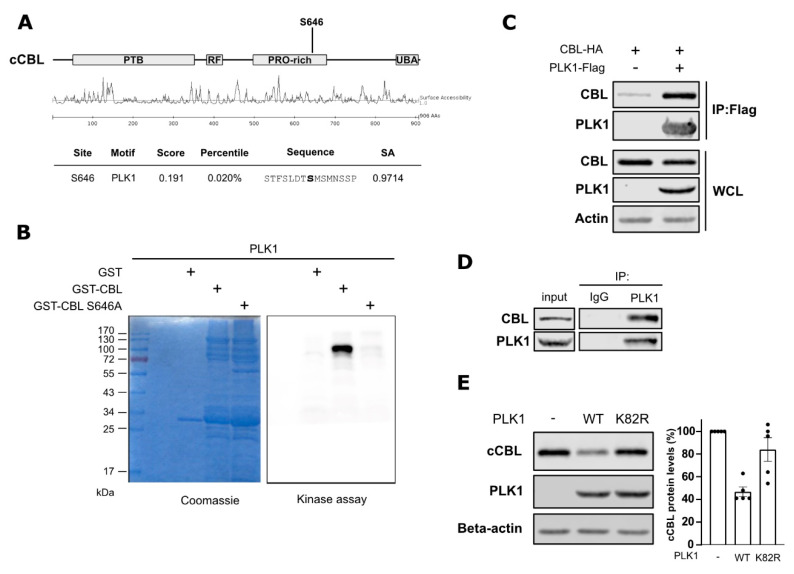
PLK1 phosphorylates c-Cbl at Serine 646. (**A**) Phosphorylation site on c-Cbl by PLK1 predicted using the MIT Scansite online tool. (**B**) Recombinant PLK1 and GST-tagged c-Cbl (wild-type or S646A mutant) were incubated together with ^32^P-γATP in kinase assay buffer. The reaction products were analyzed by SDS-PAGE and PhosphorImaging. (**C**) c-Cbl-HA and PLK1-FLAG were overexpressed in HEK293T cells for 24 h. Lysates were then subjected to immunoprecipitation using FLAG-antibodies. c-Cbl and PLK1 in whole cell lysates (WCL) and immunoprecipitates were detected using Western blotting. (**D**) Whole-cell lysates of PC9 cells were used to immunoprecipitate endogenous PLK1. WCL and immunoprecipitates were subjected to Western blotting with indicated antibodies. (**E**) PLK1-WT or PLK1-K82R (kinase-dead) were overexpressed with c-Cbl in HEK293T cells for 48 h and then processed using Western blotting. Quantification of relative c-Cbl levels is shown as mean ± SEM (*n* = 5). Dots represent individual experiments.

c-Cbl is the major E3 ubiquitin ligase of EGFR involved in the ubiquitination and lysosomal degradation of EGFR. Since we observed decreased EGFR protein levels upon PLK1 inhibition, we investigated the potential link between PLK1 and c-Cbl. Using Scansite 4.0, we found that serine 646 on c-Cbl is a candidate substrate for phosphorylation by PLK1 (Figure 2A) [16]. To test this prediction, we performed an in vitro kinase assay using recombinant PLK1 and c-Cbl in the presence of radiolabeled [γ-^32^P] ATP. We observed that PLK1 can phosphorylate c-Cbl in vitro (Figure 2B). Further, we produced recombinant c-Cbl in which serine 646 was mutated into alanine (S646A) and observed that the radioactive signal in the kinase assay was lost (Figure 2B). This result demonstrates that the c-Cbl phosphorylation by PLK1 was specifically at serine 646. To evaluate whether phosphorylation of c-Cbl can also occur in a cellular context, we assessed the ability of c-Cbl and PLK1 to interact in cells by performing immunoprecipitation studies. We first overexpressed PLK1 and c-Cbl in HEK293T cells and found that c-Cbl co-immunoprecipitated with PLK1 (Figure 2C). In a second step, we also confirmed the interaction between endogenous PLK1 and c-Cbl proteins in PC9 cells (Figure 2D). Our results indicate that PLK1 interacts with c-Cbl and directly phosphorylates it on serine 646. 

In addition, we observed that the overexpression of PLK1 induced a reduction in c-Cbl levels (Figure 2E). In contrast, the expression of a kinase-dead mutant of PLK1 (K82R mutant) did not affect c-Cbl levels in cells (Figure 2E), indicating the catalytic activity of PLK1 to be involved in the destabilization of c-Cbl. 

To check the hypothesis that these mechanisms are relevant for the effects of PLK1 inhibition on EGFR stability, we examined whether PLK1 activity or inhibition affects the interaction between c-Cbl and EGFR in cells. To do so, we overexpressed EGFR-Δ746-750 and c-Cbl in HEK293T cells, which were subsequently treated with Volasertib for three hours. Surprisingly, PLK1 activity did not affect the level of c-Cbl interacting with EGFR (Supplemental Figure S3A). Additionally, when using EGFR-WT (wild-type) in EGF-stimulated cells, Volasertib treatment did not alter the interaction between EGFR and c-Cbl, and no changes in the ubiquitination status of EGFR were observed (Supplemental Figure S3B). Moreover, we found that EGFR and PLK1 also interacted with each other, both in the overexpression and endogenous model (Supplemental Figure S4A,B). The precise functional consequences of this interaction remain to be elucidated. We thus identified a strong interaction between PLK1 and c-Cbl, but how PLK1 regulates EGFR protein levels and the impact of c-Cbl phosphorylation by PLK1 remains unclear.

### 2.3. PLK1 Inhibition Enhances Cancer Cell Sensitivity to Osimertinib in EGFR-Mutant NSCLC Cells 

**Figure 3:**
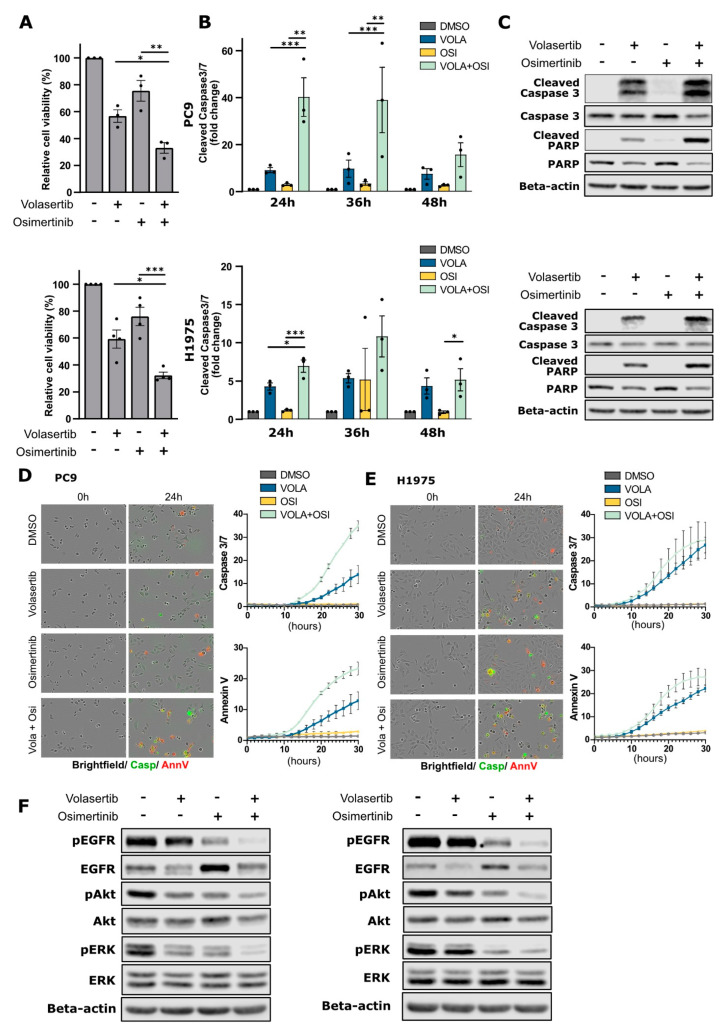
Combined EGFR and PLK1 inhibition reduces cell viability of EGFR-mutant NSCLC cells through apoptosis and reduced EGFR signaling. (**A**) Cell viability analysis of PC9 and H1975 cells treated with Volasertib (40 nM) and/or Osimertinib (10 nM) for 72 h. Bars represent mean ± SEM, *n* ≥ 3. (**B**) Caspase 3/7 cleavage analysis of PC9 and H1975 cells treated with Volasertib (40 nM) and/or Osimertinib (10nM) for the indicated timepoints. Caspase 3/7 cleavage was normalized to cell viability results for the same experiment. (**C**) PC9 and H1975 cells were treated with Volasertib (40 nM) and/or Osimertinib (10 nM) and cells were lysed after 24 h. Cell lysates were analyzed with Western blotting using the indicated antibodies. (**D**) PC9 and (**E**) H1975 cells were treated with Volasertib (40 nM) and Osimertinib (10 nM) and monitored for 30 h using the Incucyte SX5 Live-Cell Imaging system in presence of Caspase 3/7 activation (green) and Annexin V (red) dyes. Representative images of the different conditions at 24 h upon treatment are shown. Quantifications of a representative experiment show Caspase3/7 or AnnexinV positive area normalized to confluence area and relative to DMSO conditions (mean ± SD). Quantifications of two other independent repeats are shown in Supplemental Figure S5. (**F**) PC9 and H1975 cells were treated with Volasertib (40 nM) and/or Osimertinib (10 nM) and cells were lysed after 24 h. Cell lysates were analyzed with Western blotting using the indicated antibodies. Dots represent individual experiments (* *p* < 0.05; ** *p* < 0.01; *** *p* < 0.001).

The induction of EGFR degradation has shown to be an efficient strategy to improve EGFR TKI treatment in EGFR-mutant NSCLC [17]. Here, we analyzed whether the decreased EGFR protein levels obtained via PLK1 inhibition could increase the efficacy of EGFR TKIs (e.g., Osimertinib) in causing cytotoxicity in the EGFR-mutant NSCLC sensitive cell lines PC9 and H1975. We found that the combination of Volasertib and Osimertinib significantly decreased the cell viability of NSCLC cells compared to single treatments (Figure 3A). The effect was associated with an increase in caspase 3/7 cleavage in the combined treatment compared to single Volasertib or Osimertinib treatments in both PC9 and H1975 cells (Figure 3B). This was confirmed using Western blotting for apoptotic markers, such as cleaved (active) caspase-3 and cleaved PARP, which were more evident in the combined treatments (Figure 3C). In addition, upon combination therapy of Volasertib and Osimertinib in PC9 cells, we showed increased activation of caspase3/7 and extracellular phosphatidylserine (as stained by Annexin V) versus single treatments using real-time live cell monitoring, indicating enhanced apoptosis (Figure 3D,E and Supplemental Figure S5). In H1975, we observed a similar trend of increased Caspase3/7 activation and exposed phosphatidylserine in the combination; however, it was less pronounced compared to PC9 cells. Taken together, our results indicate that with the combination of Volasertib and Osimertinib leads to increased cell compared to single treatments in both cell lines.

We then examined how downstream EGFR signaling pathways were affected by combination therapy. Our preceding experiments found that Volasertib, as a single agent, reduced EGFR protein levels. In contrast, Osimertinib alone (as previously reported with the EGFR TKI afatinib [17]) increased EGFR levels (Figure 3F). Remarkably, Volasertib overcame the increase in EGFR levels instigated by Osimertinib in the combination treatment. Further, the EGFR kinase inhibition by Osimertinib treatment was enhanced by Volasertib, leading to the enhanced inhibition of effectors downstream of EGFR, as we observed a decreased activity of ERK1/2 and AKT (Figure 3F). Thus, PLK1 inhibition combined with Osimertinib decreases cell viability in EGFR-mutant NSCLC cells caused by further dampening of downstream EGFR signaling and apoptosis induction.

## 3. Discussion

EGFR TKIs have dramatically improved overall survival outcomes of EGFR-mutant NSCLC patients with a favorable therapeutic ratio due to a logarithmic higher sensitivity of the mutant versus the wild-type receptor. Unfortunately, innate and acquired resistance mechanisms ultimately result in therapy failure, leading to disease progression. Here, we demonstrated that the master cell cycle regulator, PLK1, is a valuable target for upfront combination therapy with EGFR inhibitors in NSCLC. We showed that the combination of EGFR and PLK1 inhibitors resulted in enhanced apoptosis. This decreases the pool of cancer cells surviving the primary treatment and thus may delay or avoid the emergence of acquired constitutive drug resistance. 

To date, PLK1 inhibition in combination with EGFR TKI was only studied in NSCLC cells with acquired constitutive resistance to EGFR TKI. Volasertib increased sensitivity to Erlotinib in Erlotinib-resistant cell lines via G2/M cell cycle arrest followed by apoptotic cell death [14]. In addition, PLK1 inhibition also induces apoptosis in Osimertinib-resistant cells [15]. 

The current work shows that PLK1 and EGFR co-targeting is also a valuable option in treatment-naïve, EGFR-mutant NSCLC before constitutive resistance develops. Combined PLK1 and EGFR inhibition as initial treatment for EGFR-mutant NSCLC might thus prevent the emergence of an array of different constitutive resistance mechanisms rather than remedy this complexity later. Moreover, we demonstrated that, apart from the inhibition of the cell cycle, PLK1 inhibition reduces EGFR protein levels, which contributes to the observed cooperative therapeutic efficacy. The two cell lines used in our study, PC9 and H1975, harboring EGFR del746-750 and L858R/T790M mutation types, respectively, show a similar effect to PLK1 inhibition. While these two cell lines represent the two most frequent EGFR mutations found in NSCLC, further clinical studies are required to establish the role of dual targeting of PLK1 and EGFR in a clinical setting and in the context of other EGFR mutations.

The clinical successes of PLK1 inhibitors as single agents are limited; PLK1 monotherapy resulted in partial responses in only a few patients. Additionally, in NSCLC patients, the efficacy of PLK1 inhibitors was low, although these agents are generally well-tolerated [18,19]. The current work gives a new incentive for the clinical investigation of combinatorial strategies in EGFR-mutated NSCLC patients. 

Previous research also showed that the degradation of EGFR is a valuable strategy to diminish the survival of EGFR-mutant NSCLC cells. We and others have shown that direct EGFR downregulation in combination with EGFR TKIs enhances therapeutic efficacy by an additive effect on growth inhibition and induction of apoptosis [20]. We also showed that co-targeting of USP13 increased the sensitivity to EGFR-targeted therapies, by enhanced EGFR degradation [17]. The small molecule T315 also sensitizes for EGFR TKI Afatinib via the induction of EGFR degradation [21]. Proteolysis-targeting chimeras (PROTACs) that target EGFR for degradation are being explored and show good anti-tumor responses in pre-clinical studies [22]. In this study, we demonstrate that targeting PLK1 through pharmacological inhibition or siRNA knockdown forms a novel strategy to decrease EGFR protein levels. EGFR degradation eliminates the receptor’s enzyme-independent functions, which are not inhibited by EGFR TKI, and results in long-lasting signaling inactivation. The kinase-independent (KID) functions of EGFR promote cancer cell survival by supporting the import of nutrients, promoting autophagy, and inhibiting apoptosis [23]. Moreover, the scaffolding functions of EGFR provoke kinome re-wiring upon EGFR TKI treatment to restore oncogenic signaling [24]. We speculate that the removal of the KID functions of EGFR by induction of EGFR degradation underlies the enhanced efficacy of the combination of PLK1 and EGFR inhibitors.

We identified c-Cbl, the major E3 ubiquitin ligase responsible for EGFR ubiquitination that provokes receptor degradation, as a novel substrate of PLK1. We showed for the first time the phosphorylation of c-Cbl on serine 646 by PLK1. Multiple serine, threonine, and tyrosine phosphorylation sites on c-Cbl have been identified to regulate c-Cbl function. Other neighboring serine sites on c-Cbl, namely S619, S623, S639, and S642, were previously shown to be phosphorylated by protein kinase C and prevent the phosphorylation of tyrosine residues on c-Cbl and the interaction of c-Cbl with SH2-containing signaling proteins [25]. C-Cbl tyrosine phosphorylation on Y307, Y337, Y371 and Y368 is associated with increased ubiquitination activity [26]. In particular, Y371 phosphorylation enhances binding to activated EGFR and induces an altered conformation of c-Cbl that removes the negative regulation on its RING domain, allowing for substrate ubiquitination. Both Y368 and Y371 are critical phosphorylation sites for c-Cbl ubiquitin ligase activity. Tyrosine phosphorylation at the C-terminus of c-Cbl at Y700, Y731, and Y774 serves as docking sites for SH2 domain-containing proteins and is necessary for the interaction of c-Cbl with CIN85, which mediates endocytosis and the downregulation of EGFR [27,28]. Lastly, c-Cbl double phosphorylation on T640 and S866 by ATM causes c-Cbl stabilization [29]. The downstream biological effects of c-Cbl serine 646 phosphorylation remain to be explored in further studies. Potentially, c-Cbl serine 646 phosphorylation has similar functions to the nearby phosphorylation sites. It remains to be addressed whether the lack of c-Cbl S646 phosphorylation is associated with the degradation of EGFR upon PLK1 inhibition. 

Contradictory to the c-CBL-dependent degradation of EGFR, we observed a decrease in c-CBL levels along with a decline in EGFR levels upon PLK1 inhibition. The expected c-CBL stabilization is offset by the co-degradation of CBL with its substrate EGFR, as shown by Ettenberg et al. [30].

PLK1 may affect EGFR stability in an alternative way, as we show that mutant EGFR and PLK1 interact with each other. The interaction of PLK1 with wild-type EGFR was previously identified in a high-throughput mass spectrometry experiment. This study showed increased interaction of PLK1 with EGFR in pre-and post-internalization steps upon EGF stimulation [31]. We confirmed the interaction of mutant EGFR and PLK1 in co-immunoprecipitation experiments. This suggests that PLK1 may directly affect EGFR and that the PLK1-induced decrease in EGFR protein levels is via a c-Cbl-independent mechanism. A follow-up study involving (phospho)proteomics to determine changes in phosphorylation status and interactome of EGFR instigated by PLK1 is needed to unravel the interplay of PLK1 and EGFR. 

Other E3 ubiquitin ligase proteins such as ZNRF1 [32] and CHIP [33] participate in EGFR ubiquitination. We demonstrated that the total ubiquitination of wild-type EGFR in the presence of c-Cbl was not impacted by the inhibition of PLK1. 

## 4. Materials & Methods

### 4.1. Cell Lines and Drugs

PC9 (Sigma-Aldrich, Saint-Louis, MO, USA) and H1975 (ATCC, Manassas, VA, USA) cells were both cultured in RPMI-1640 medium (21875-034, Gibco, Waltham, MA, USA); HEK293T (ATCC) cells were cultured in DMEM (12491-015, Gibco). Both media were supplemented with 10% FBS (758093, Greiner, Monroe, NC, USA) and 1% penicillin-streptomycin. Cells were kept at 37 °C in a 5% CO_2_ atmosphere under sterile conditions. PLK1 and EGFR inhibitors were bought from Selleckchem (Houston, TX, USA): Volasertib (S2235), RO3280 (S7248), and Osimertinib (S7297). The final concentrations used are mentioned in the figure legends. Cycloheximide (C7698) was purchased from Sigma.

### 4.2. SiRNA and DNA Plasmids

PLK1 siRNA sequences (set of four, LQ-003290-00-0002) were obtained from Horizon discovery (Waterbeach, UK). The cells were transfected with pooled siRNAs using Lipofectamine^®^ RNAiMAX (13778-030, Thermo Fisher, Waltham, MA, USA) according to the manufacturer’s protocol. Final siRNA concentrations were 6 nM.

The following plasmids were used: pcDNA4-EGFR-(746-750)-myc-His B; HA-cCbl (both kindly provided by Prof. Kwang Y. Lee (Chonnam National University, Republic of Korea)); and pCMV3-C-FLAG-PLK1 (HG10676-CF, Bio Connect life sciences). Site-directed mutagenesis was used to generate cCBL-S646A (forward primer: 5′-gctattcatactcatggcggtatccagactgaacg-3′ and reverse primer: 5′-cgttcagtctggataccgccatgagtatgaatagc-3′) and PLK1-K82R (forward primer: 5′-GACTTAGGCACAATCCTGCCCGCGAACACCT-3′ and reverse primer: 5′-AGGTGTTCGCGGGCAGGATTGTGCCTAAGTC-3′).

### 4.3. Quantitative Real-Time PCR

Total mRNA extraction was performed using the Nucleospin RNA plus kit (#740984, Macherey-Nagel, Bethlehem, PA, USA) according to the manufacturer’s protocol. First-strand cDNA was synthesized using SuperScript™ II Reverse Transcriptase (#18064014, ThermoFisher, Waltham, MA, USA). Target and reference genes were quantified in duplicates on a LightCycler^®^480 (Roche, Basel, Switzerland) using SYBR Green I Mastermix (#04707516001, Roche). The geometric means of the housekeeping genes TBP and SDHA were used to normalize input cDNA.

### 4.4. Western Blotting

Cells were lysed using a buffer containing 1% Triton X-100, 20 mM Tris-HCl (pH = 7.5), 150 mM NaCl, 1 mM EDTA, 2.5 mM sodium-pyrophosphate, and 1 mM sodium orthovanadate, supplemented with 1% phosphatase inhibitors (#P5726, Sigma-Aldrich) and 1% protease inhibitors (#P8340, Sigma-Aldrich). Protein concentrations were calculated using the Bradford protein assay kit (Bio-Rad, Hercules, CA, USA). Equal amounts of proteins (10–30 µg) were loaded and separated by SDS-PAGE using 10–15% resolving acrylamide gels. Proteins were transferred overnight onto nitrocellulose membranes. After blocking the membranes with 5% non-fat milk, primary antibodies were diluted in 5% bovine serum albumin (BSA) containing Tris-buffered saline with Tween-20 (TBST) and incubated overnight at 4 °C. The appropriate secondary infrared-conjugated antibodies (LI-COR, Lincoln, NE, USA) were incubated for one hour at room temperature and protected from light. Detection was performed using the LI-COR Biosciences Odyssey^®^ Fc Imaging System and analyzed with the Image Studio™ software 4.0 (LI-COR). Original Western blots can be found in Supplemental Figure S6.

Primary antibodies used for Western blotting in this study were: PLK1 (#4513); caspase-3 (#9665); PARP (#9532); phospho-EGFR (Tyr1068, #3777); total ERK (#4695); phospho-ERK1/2 (Thr202/Tyr204, #4370); total Akt (#9272); and phospho-Akt (Ser473, #4058) from Cell Signaling Technology (Danvers, MA, USA); total EGFR (AMAb90816), c-Cbl (HPA027956), and β-actin (A1978) were from Sigma-Aldrich.

### 4.5. Co-Immunoprecipitation

Cells were lysed 24 h after transfection with the indicated plasmids using Triton X-100 containing lysis buffer. Equal amounts of proteins were subjected to immunoprecipitation, and 20 µg of each condition were saved as control inputs. Immunoprecipitation was performed for 3 h at 4 °C using anti-EGFR antibodies (GRO1, Sigma-Aldrich) combined with protein G-coupled Sepharose (17-0618-01, GE Healthcare, Chicago, IL, USA) or anti-FLAG coupled agarose (A2220, Sigma-Aldrich). After incubation, the beads were washed three times using ice-cold PBS. Proteins were eluted by adding a 2X SDS-containing loading buffer and subjected to Western blotting. 

### 4.6. Cell Viability

Cells were plated at a density of 350–500 cells in a 384-well plate. After culturing them for 24 h, Osimertinib and Volasertib were added to the cells at the indicated doses. After an incubation time of 72 h, cell viability was measured using the CellTiter-Glo^®^ luminescent kit (G7571, Promega, Madison, WI, USA) with the help of a Spectromax^®^ M3 (Molecular Devices, San Jose, CA, USA).

### 4.7. Apoptosis Assay Caspase-Glo 3/7

Cells were seeded at a density of 4000–5000 cells in a 96-well plate. After 24 h, the cells were drug-treated for the indicated timepoints (24 h, 36 h, or 48 h). Cell viability and cleaved caspase 3/7 levels were measured with CellTiter-Glo^®^ luminescent kit (G7571, Promega) and Caspase-Glo^®^ 3/7 assay kit (G8091, Promega), respectively. Caspase 3/7 signals were normalized to the total number of cells using CellTiter-Glo signals of the same conditions.

### 4.8. Apoptosis Assay Incucyte

Cells were seeded using CaCl_2_ (1 mM) supplemented medium at a density of 4000–5000 cells in a 96-well plate. After 24 h, together with the compounds, the Incucyte^®^ Annexin V (4641, Sartorius, Göttingen, Germany) and Caspase-3/7 (4440, Sartorius) dyes were added to the cells according to the manufacturer’s protocol. To monitor Caspase-3/7 and AnnexinV signals over time, the plate was installed in the Incucyte^®^ SX5 Live-Cell Analysis System (Sartorius) and images were acquired every two hours. Data analysis was performed using Incucyte software (2022B). Red (AnnexinV) and green (Caspase-3/7) fluorescence area was normalized to cell confluence area.

### 4.9. Recombinant Proteins and In Vitro Kinase Assay

HA-c-Cbl (kindly provided by Prof. Kwang Y. Lee (Chonnam National University, South Korea)) was used for subcloning of c-Cbl to pGEX4T2 to generate pGEX4T2-c-Cbl (WT) (forward primer: 5′-AGTGGATCCATGGCCGGCAACGTGAAGAAGAG-3′ and reverse primer: 5’-GTAGCGGCCGCCTAGGTAGCTACATGGGCAGGAG-3’). pGEX4T2-c-Cbl-S646A was created by site-directed mutagenesis. pGEX4T2-c-Cbl and pGEX4T2-c-Cbl-S646A were transformed in BL21-DE3 E. coli. When OD reached 0.7–0.8, protein synthesis was induced by adding isopropyl β-D-1-thiogalactopyranoside (IPTG). After an additional incubation of five hours, bacteria pellets were saved for protein purification. For the purification, pellets were resuspended in PBS containing protease inhibitors and lysozyme (1 mg/mL), incubated on ice for 20 min and followed by sonification. Triton-X was added at a final concentration of 1%. The lysate was centrifuged at 15,000 rpm for 25 min. Next, the supernatant was incubated with glutathione sepharose beads (17-0756-01, GE Healthcare) for 1 h at 4 °C. The beads were washed twice with PBS and once with 50 mM Tris-HCl pH = 8.0. The proteins were finally eluted using 50 mM glutathione (GE Healthcare) in 50 mM Tris-HCl pH = 8.0. Overnight dialysis was performed in a buffer containing 20 mM Tris-HCl pH = 7.6, 50 mM NaCl, 5 mM EDTA, 1 mM DTT, and 5% glycerol. SDS-PAGE and Coomassie blue staining confirmed the molecular weight and integrity of the recombinant proteins.

For kinase reactions in vitro, a few micrograms (1–5 μg) of c-Cbl (WT) or c-Cbl-S646A were incubated with recombinant PLK1 (V2841, Promega) in the presence of 50 μM unlabeled ATP and 0.5 μL of ^32^P-labeled γATP (Perkin Elmer, Waltham, MA, USA, BLU002250UC) in a kinase buffer (V2841, Promega). The reaction was incubated for 30 min at 30 °C, stopped by adding 2X sample buffer, and finally loaded onto an SDS-PAGE. After running, the gels were stained with Coomassie blue, dried, exposed and visualized with the help of a PhosphorImager (BioRAD, Hercules, CA, USA).

### 4.10. Statistics

Graphs are represented by means and standard error of the mean (SEM). Statistical analysis was performed using GraphPad Prism. The student’s *t*-test was used to assess the significance between two groups and Brown–Forsythe ANOVA for experiments with three experimental conditions. Two-way ANOVA with Tukey post-hoc analysis was performed on the cell viability experiments using the combination treatment. *p*-value less than 0.05 was considered statistically significant (* *p* < 0.05; ** *p* <0.01; *** *p* <0.001; **** *p* <0.0001).

## 5. Conclusions

In conclusion, we found that targeting PLK1 through pharmacological inhibition or siRNA-mediated silencing results in reduced mutant EGFR protein levels. The co-targeting of PLK1 sensitizes EGFR-mutant NSCLC cells to EGFR TKI in vitro. The role of PLK1 on EGFR and c-Cbl stabilization provides a new rationale to evaluate the combination of EGFR and PLK1 inhibitors in the clinic. 

## Data Availability

Data are contained within the article or Supplementary Materials.

## Supplementary Materials

The following are available online at https://www.mdpi.com/article/10.3390/cancers15092589/s1: Figure S1: PLK1 inhibition decreases EGFR in time- and concentration-dependent manner. Figure S2: PLK1 inhibitors and Nocodazole induce similar G2/M cell cycle arrest. Figure S3: Volasertib does not alter the interaction between EGFR and c-Cbl. Figure S4: PLK1 interacts with EGFR. Figure S5: Combined PLK1 and EGFR inhibition results in apoptosis induction. Figure S6: Original uncropped Western blots.
