# Supplementary material for: Inhibition of PLK1 Destabilizes EGFR and Sensitizes EGFR-Mutated Lung Cancer Cells to Small Molecule Inhibitor Osimertinib
Source: Cancers (Basel). 2023 May 2;15(9):2589. doi: 10.3390/cancers15092589 (PMC10177332; doi:10.3390/cancers15092589)
Supplement: Supplementary file 1 [file cancers-15-02589-s001.zip › cancers-2337039-supplementary.pdf]

SUPPLEMENTAL INFORMATION

Inhibition of PLK1 Destabilizes EGFR and Sensitizes EGFR-Mutated Lung Cancer Cells to Small Molecule Inhibitor Osimertinib

Carolien Eggermont [1], Gustavo J. Gutierrez [2], Jacques De Grève [1,3] and Philippe Giron [1,3,*]

Table of contents:

Supplemental Figure S1 - PLK1 inhibition decreases EGFR in time- and concentration dependent manner 2

Supplemental Figure S2 - PLK1 inhibitors and nocodazole induce similar G2/M cell cycle arrest. 3

Supplemental Figure S3 – Volasertib does not alter the interaction between EGFR and c-Cbl 4

Supplemental Figure S4 – PLK1 interacts with EGFR. 5

Supplemental Figure S5 – Combined PLK1 and EGFR inhibition results in apoptosis induction 6

Supplemental Figure S6 - Original uncropped western blots 7

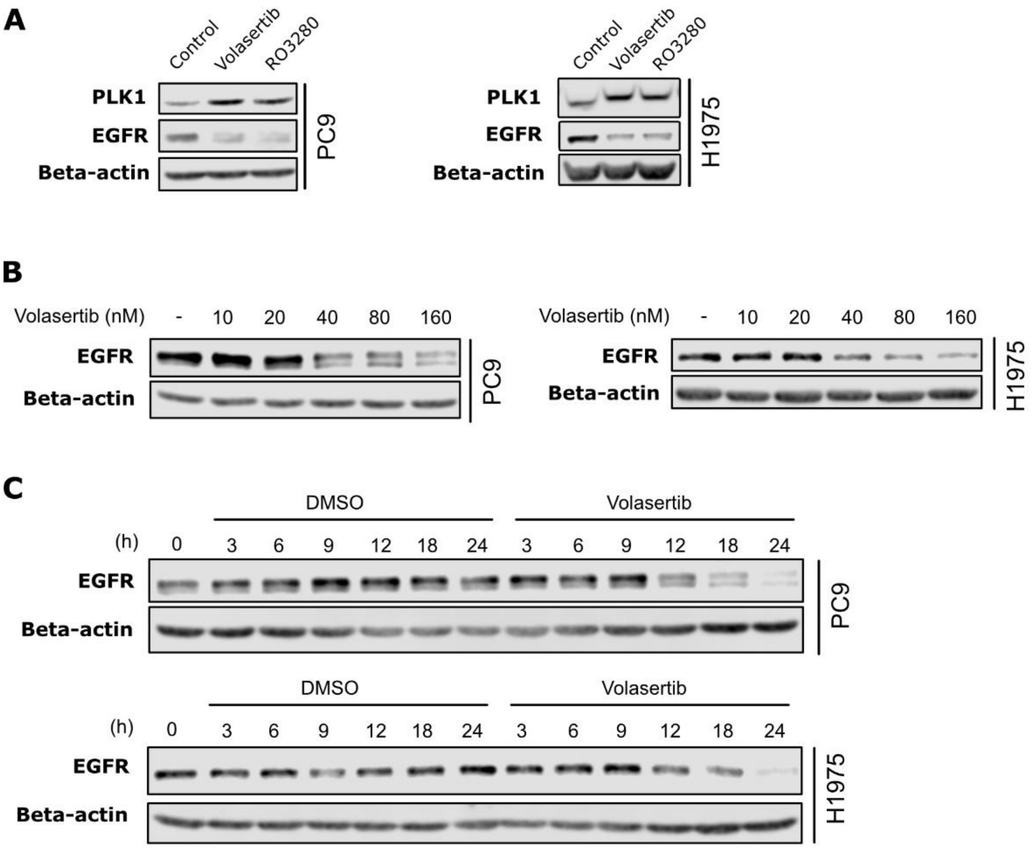

Supplemental Figure S1. PLK1 inhibition decreases EGFR in time- and concentration dependent manner. *(A) PC9 and H1975 cells were treated with 80nM Volasertib or RO280 for 24h before lysis. Lysates were analyzed by western blotting with the indicated antibodies. (B) PC9 and H1975 cells were treated with vehicle or 10, 20, 40, 80 or 160nM volasertib for 24h. Lysates were analyzed by western blotting with indicated antibodies. (C) PC9 and H1975 cells were treated with vehicle or volasertib (80nM), lysed after 3, 6, 9, 12, 18, or 24 hours incubation, and then analyzed by western blotting.*

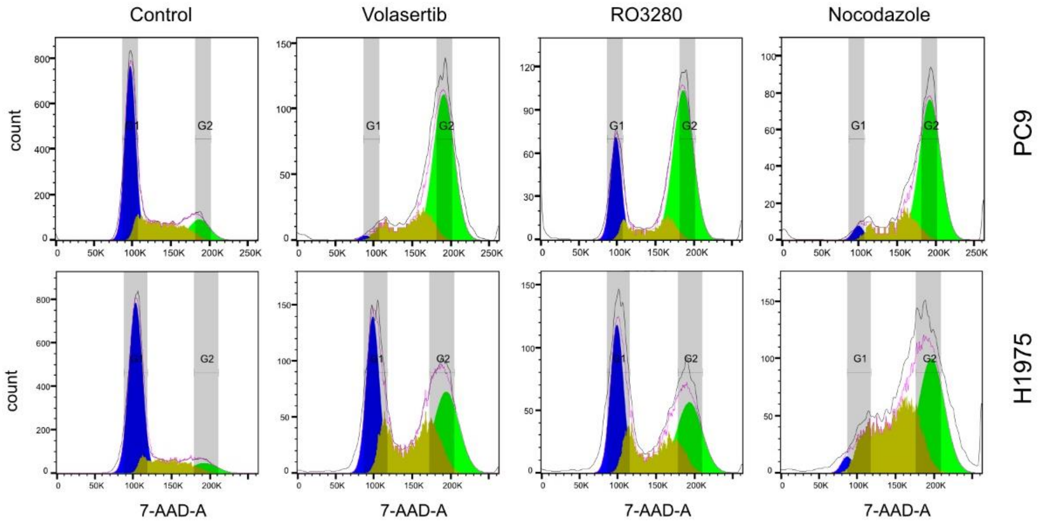

Supplemental Figure S2. PLK1 inhibitors and nocodazole induce similar G2/M cell cycle arrest. *PC9 and H1975 cells were treated with volasertib (80nM), RO3280 (80nM) or nocodazole (200ng/ml) for 24h. Cells were then stained with 7-AAD and their cell cycle profile was analyzed by flow cytometry.*

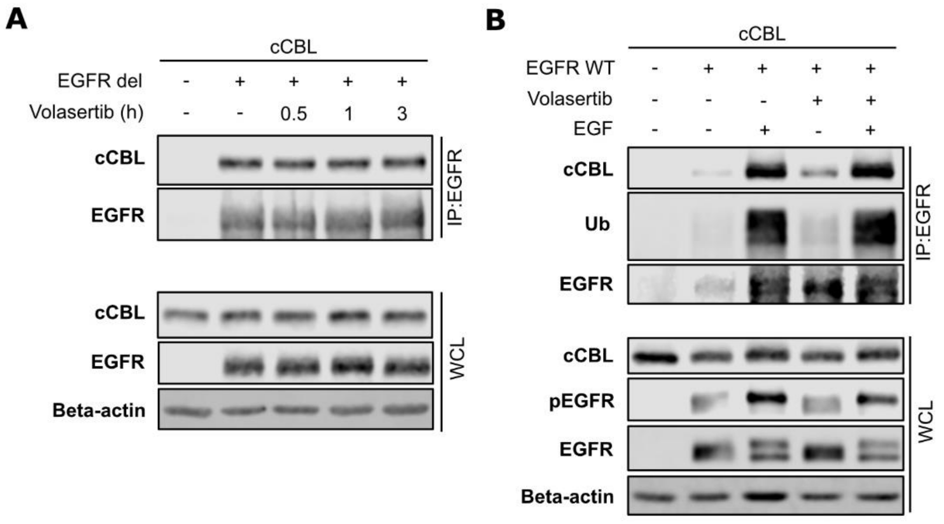

Supplemental Figure S3. Volasertib does not alter the interaction between EGFR and c-Cbl. *(A) c-Cbl and EGFR-Δ746-750 mutant were overexpressed in HEK293T cells and treated for 30min, 1 hour or 3 hours with volasertib before lysis. Immunoprecipitation was performed using anti-EGFR antibodies and immunoprecipitates were subjected to western blotting. (B) c-Cbl and EGFR-WT were overexpressed in HEK293T cells and treated for 3 hours with volasertib. Before lysis, cells were stimulated with EGF ligand (100ng/ml) for 15 minutes. Immunoprecipitation was performed using anti-EGFR antibodies and immunoprecipitates were subjected to western blotting with indicated antibodies.*

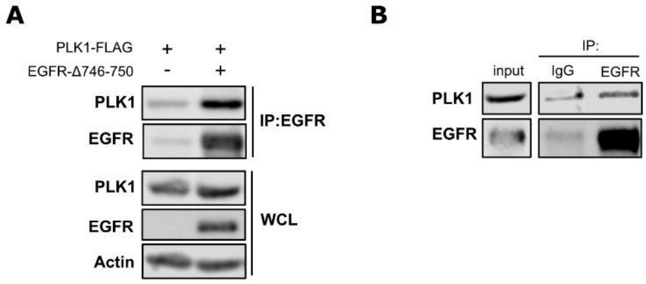

Supplemental Figure S4. PLK1 interacts with EGFR. *(A) EGFR-Δ746-750 and PLK1-FLAG were overexpressed in HEK293T cells and cells were lysed after 24 hours of incubation. Lysates were subjected to immunoprecipitation using EGFR antibodies. EGFR and PLK1 in whole cell lysates (WCL) and immunoprecipitates were detected using western blotting analysis. (B) Whole-cell lysates of PC9 cells were subjected to immunoprecipitation using control or EGFR antibodies. WCL and immunoprecipitates were subjected to western blotting with the indicated antibodies.*

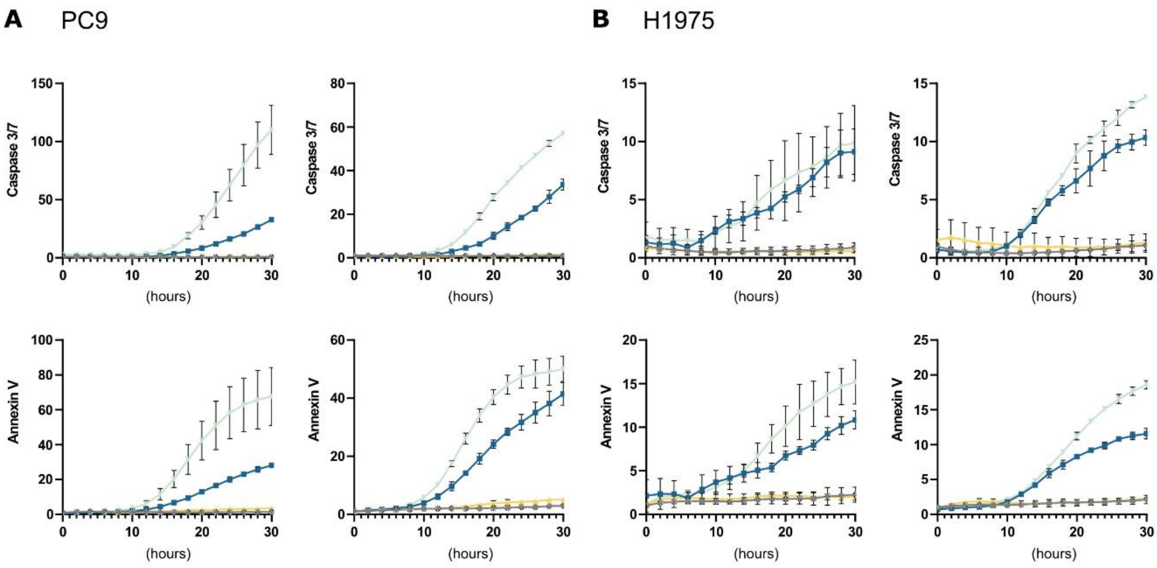

Supplemental Figure S5. Combined PLK1 and EGFR inhibition results in apoptosis induction. *(A) PC9 and (B) H1975 cells were treated with Volasertib (40nM) and Osimertinib (10nM) and monitored for 30 hours using the Incucyte SX5 Live-Cell Imaging system in presence of Caspase 3/7 activation and Annexin V dyes. Quantifications of two individual replicates show Caspase3/7 or AnnexinV positive area normalized to confluence area and relative to DMSO conditions (mean±SD).*

Supplemental Figure S6:

Uncropped Western blot from Figure 1A

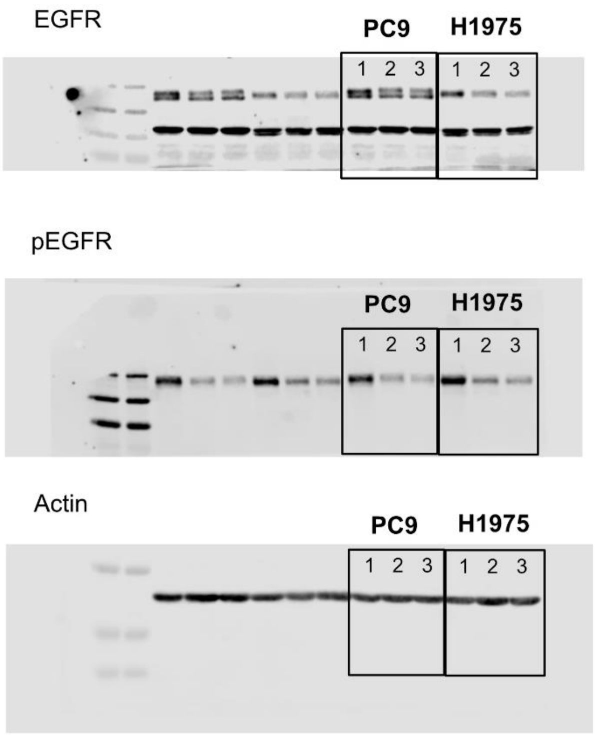

Lanes:

(1) Control treatment

(2) Volasertib 80nM

(3) RO3280 80nM

Uncropped Western blot from figure 1C

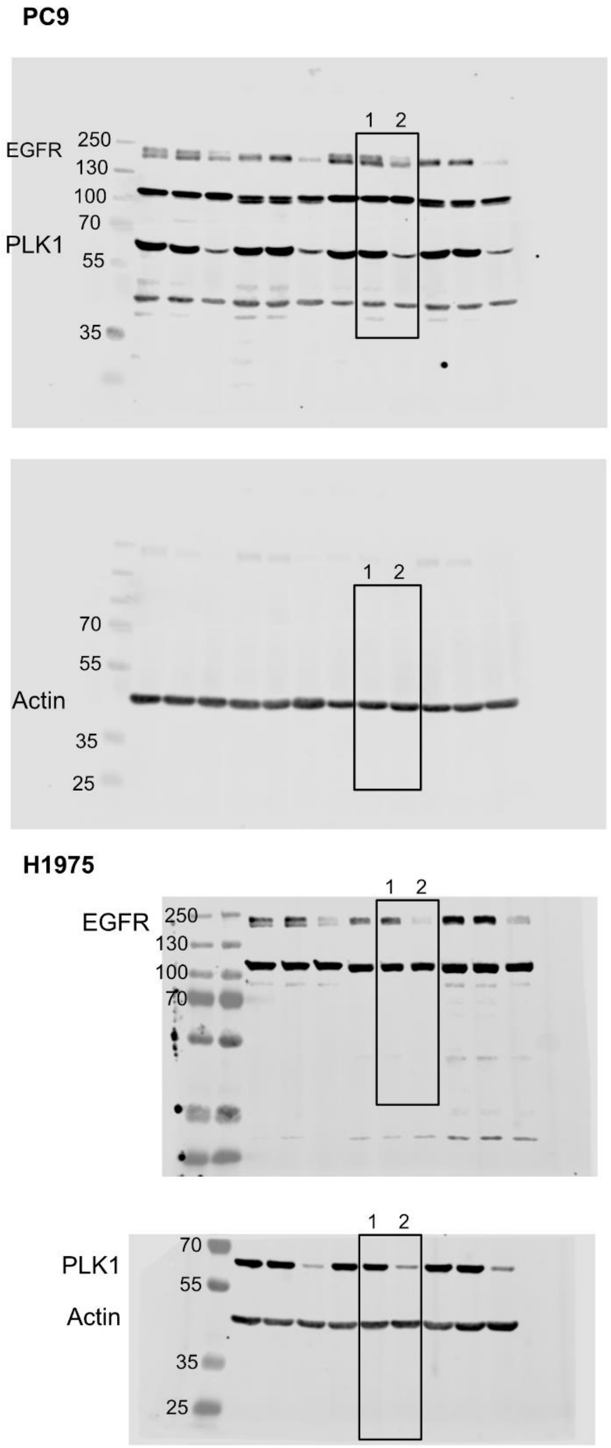

Lanes:

1. *(1) Control siRNA transfections*
2. *(2) PLK1 siRNA transfection*

Uncropped Western blot from figure 1D

PC9: EGFR

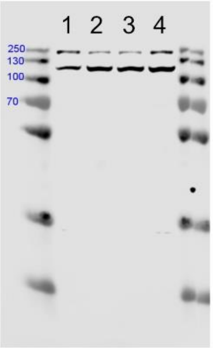

PC9: Actin

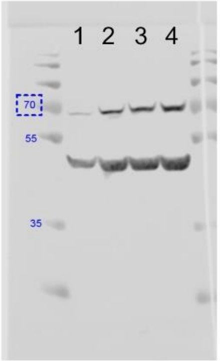

H1975: EGFR

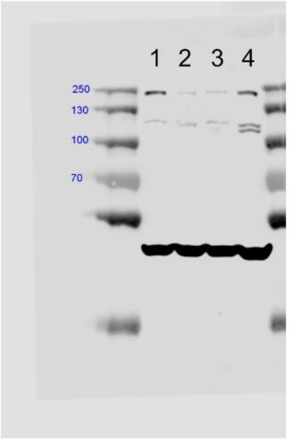

H1975: Actin

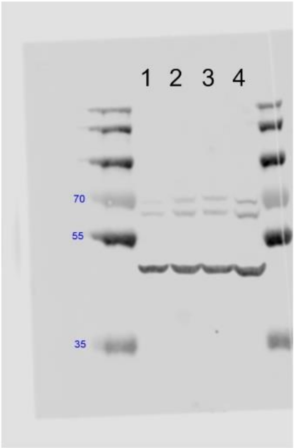

Lanes:

(1) *Control treatment*

(2) *Volasertib 80nM*

(3) *RO3280 80nM*

(4) *Nocodazole 200ng/ml*

Uncropped Western blot from figure 1F

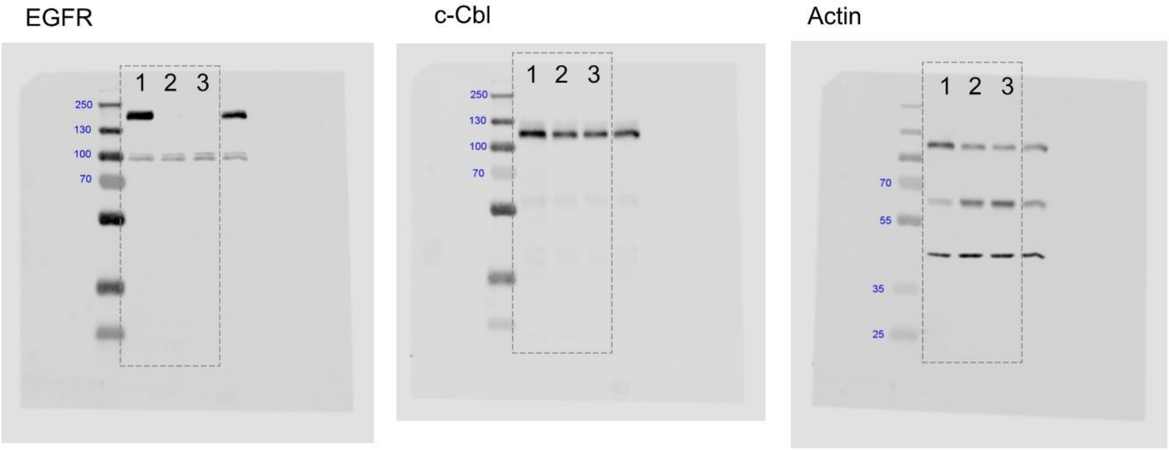

Lanes:

(1) Control

(2) Volasertib

(3) RO3280

Uncropped Western blot from figure 1G

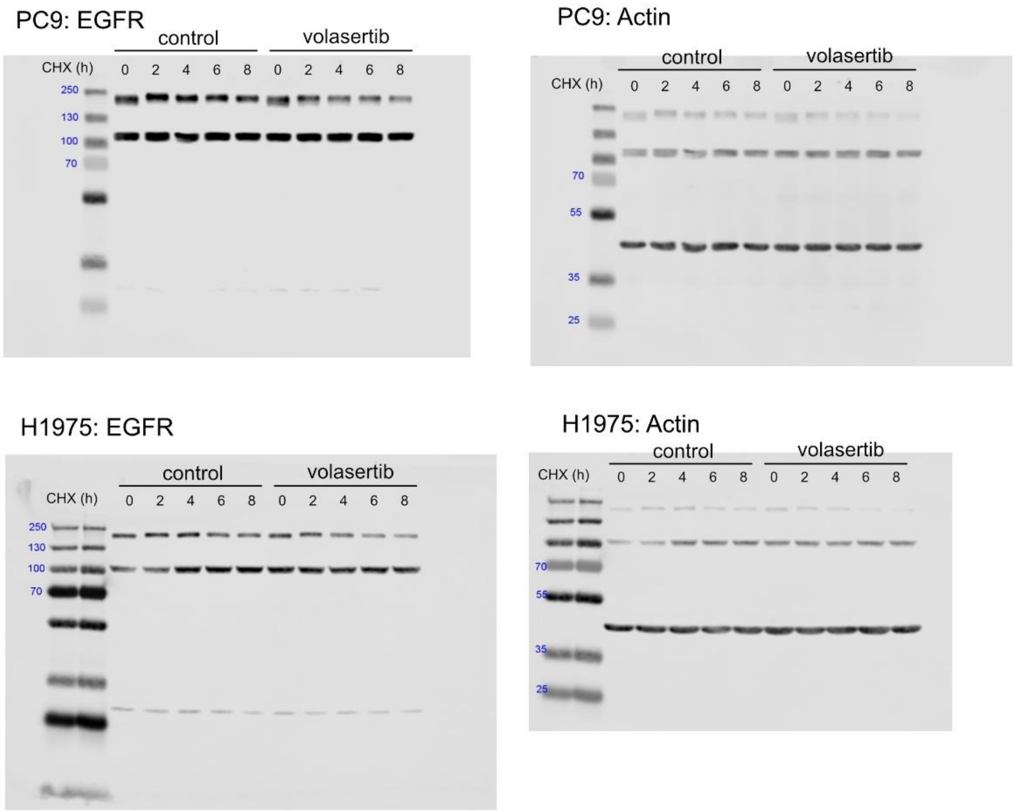

Uncropped Western blot from figure 2C

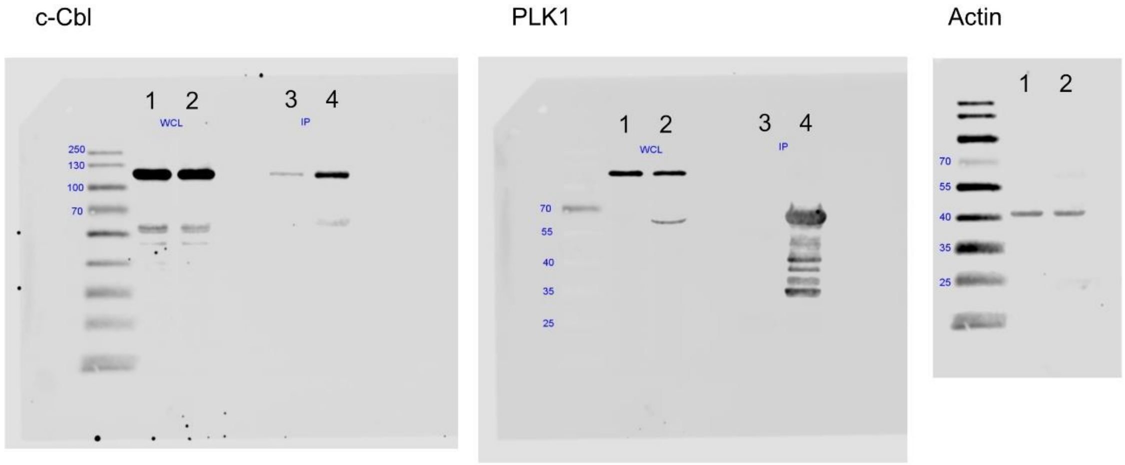

Lanes:

(1) *WCL: c-Cbl-HA*

(2) *WCL: c-Cbl-HA + PLK1-FLAG*

(3) *IP FLAG: c-Cbl-HA*

(4) *IP FLAGL: c-Cbl-HA + PLK1-FLAG*

Uncropped Western blot from figure 2D

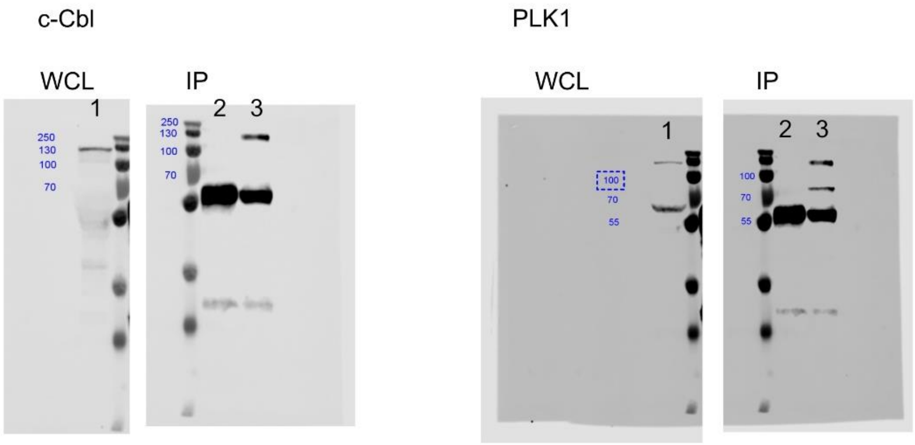

Lanes:

(1) *WCL*

(2) *IP igG*

(3) *IP PLK1*

Uncropped Western blot from figure 2E

c-Cbl

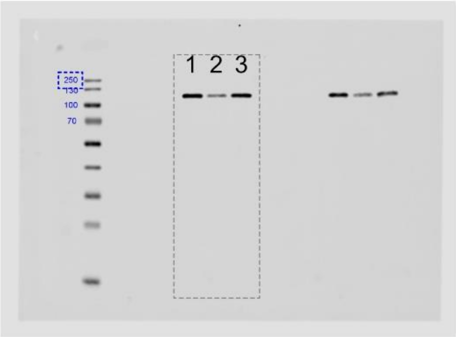

PLK1

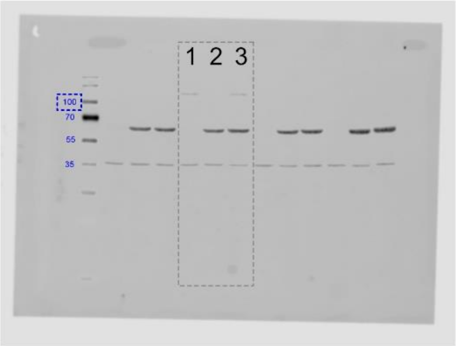

Actin

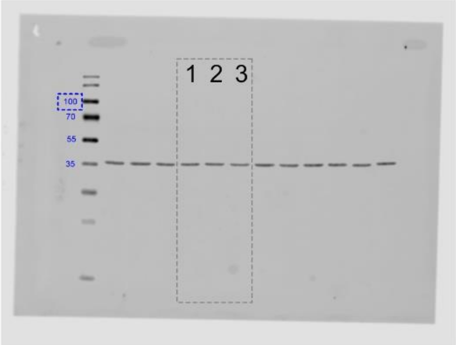

Lanes:

(1) C-Cbl +EV
(2) C-Cbl+ PLK1-WT
(3) C-Cbl+ PLK1-K82R

Uncropped Western blot from figure 3B

Casp3+Cleaved Casp3_High Exp

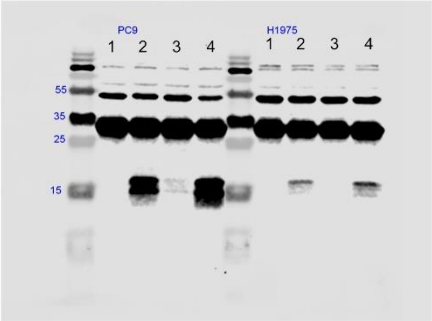

Casp3+Cleaved Casp3_Low Exp

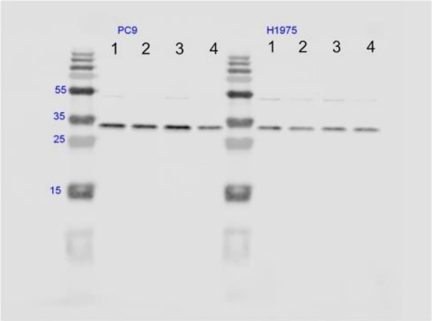

PARP+Cleaved PARP

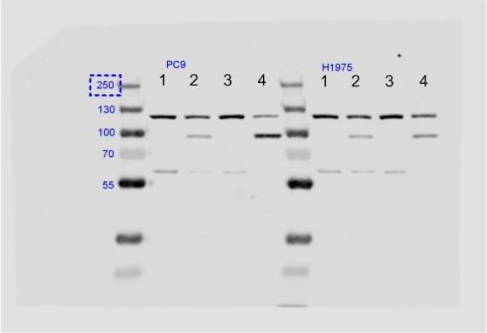

Actin

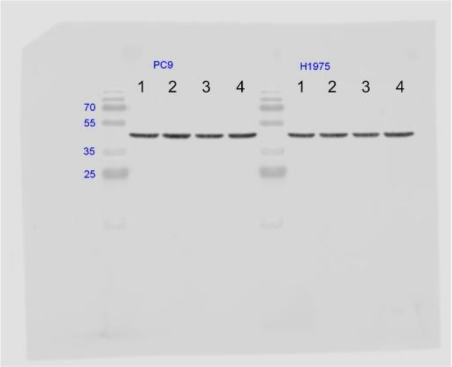

Lanes:

(1) Control

(2) Volasertib

(3) Osimertinib

(4) Volasertib + osimertinib

Uncropped Western blot from figure 3C

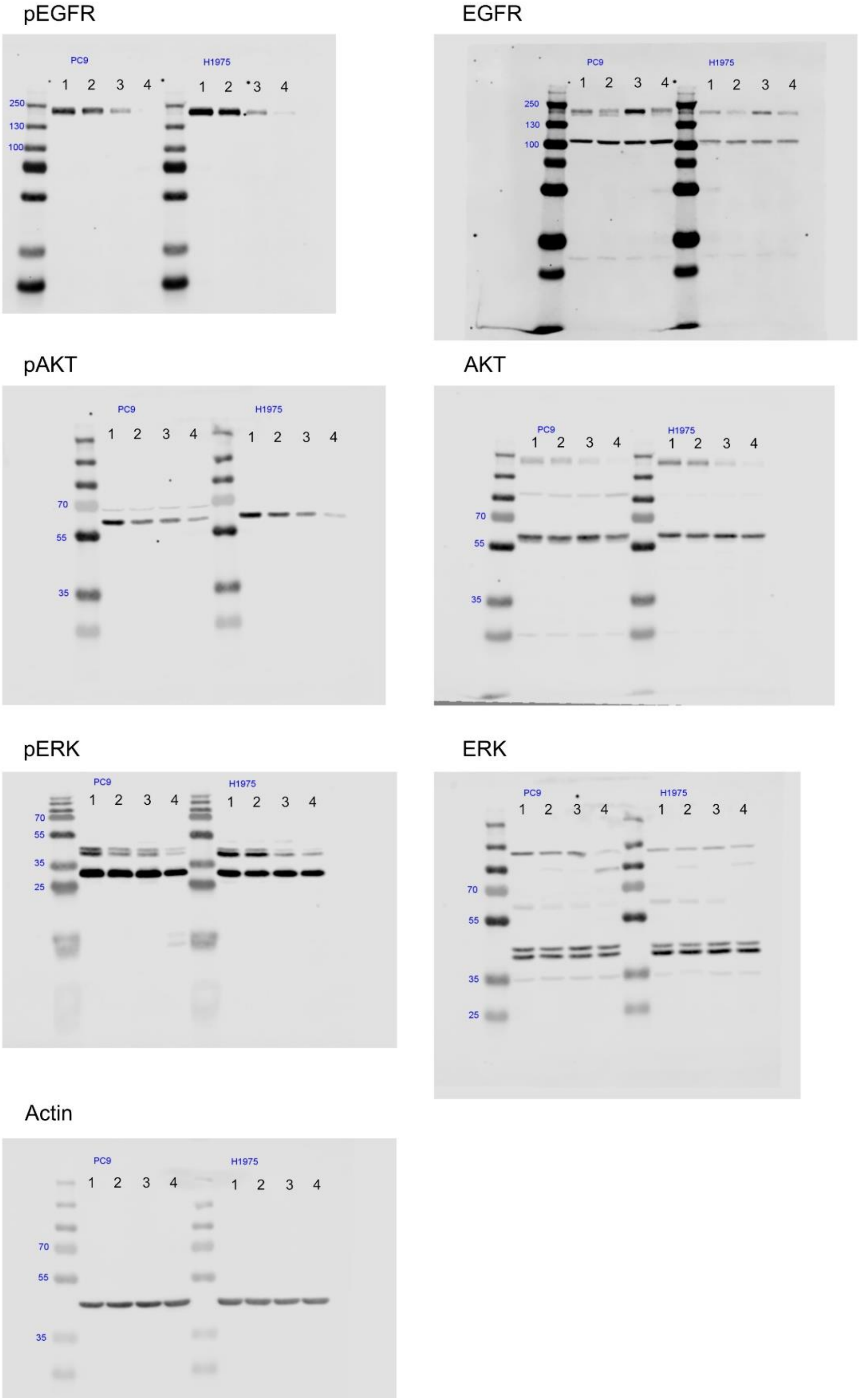

Lanes:

1. *Control*
2. *Volasertib*
3. *Osimertinib*
4. *Volasertib + osimertinib*

Uncropped Western blot from supplemental figure 1A

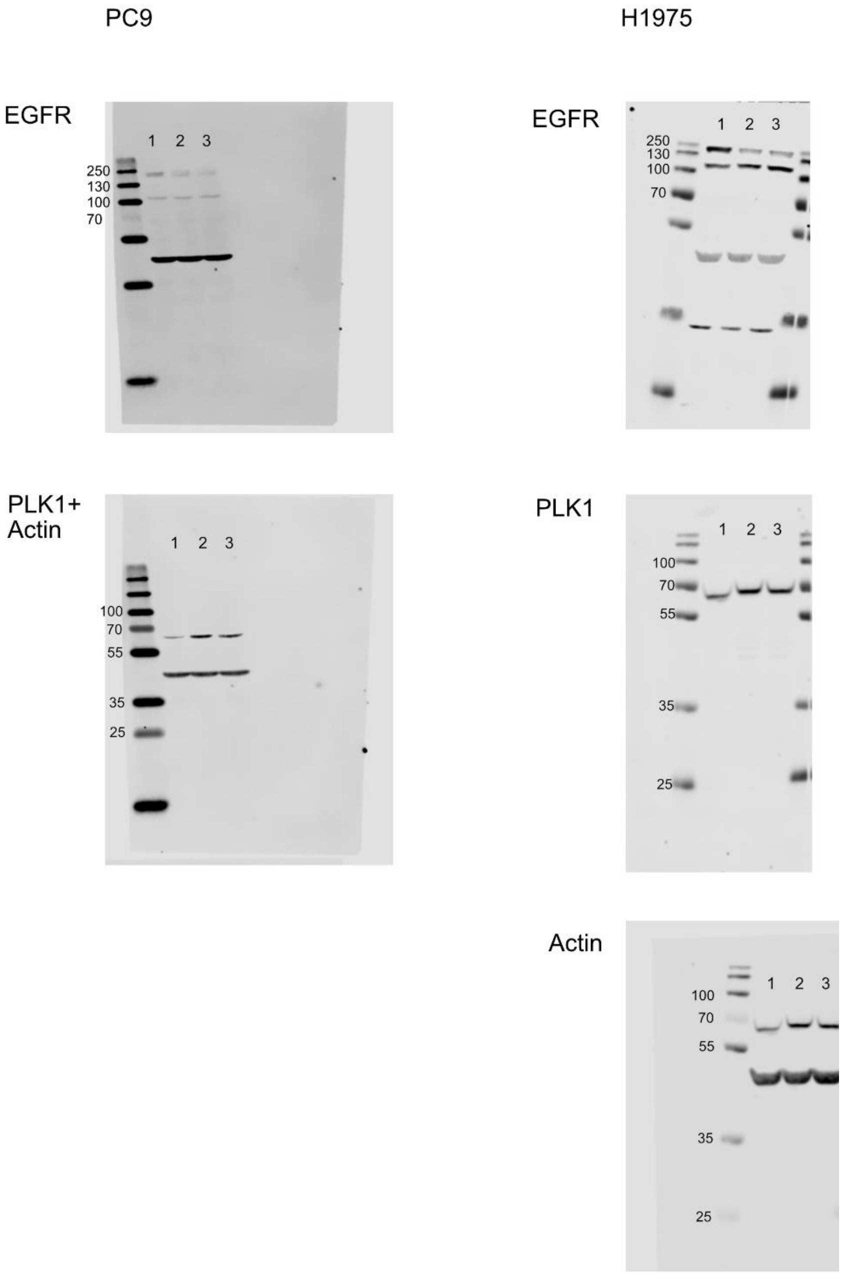

Lanes:

(1) Control

(2) Volasertib

(3) RO3280

Uncropped Western blot from supplemental figure 1B

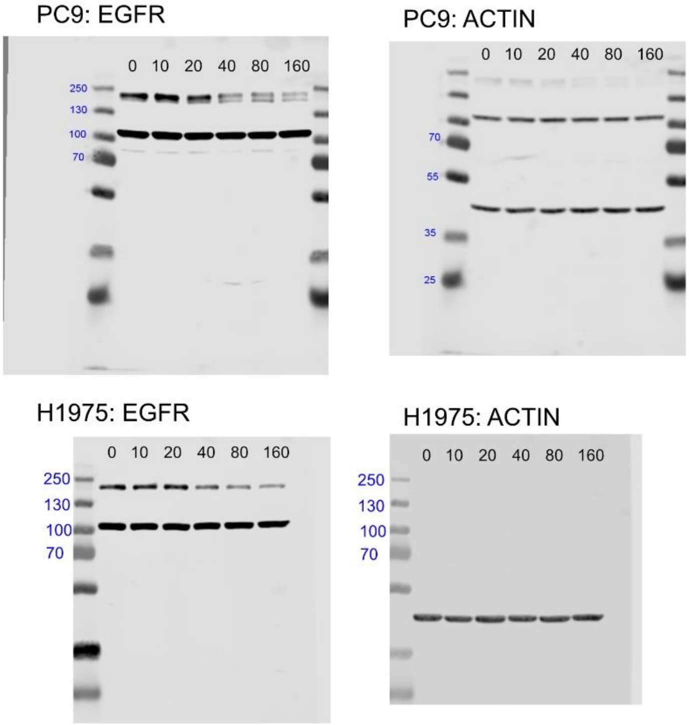

Uncropped Western blot from supplemental figure 1C

PC9: EGFR

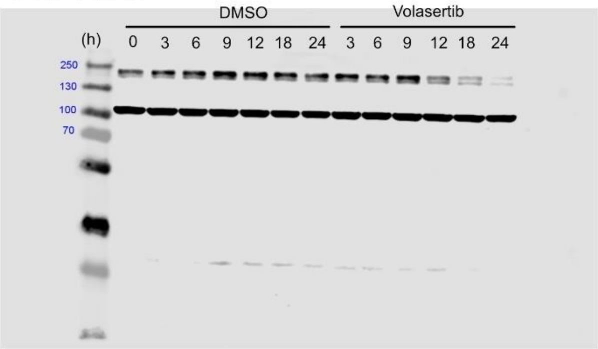

PC9: ACTIN

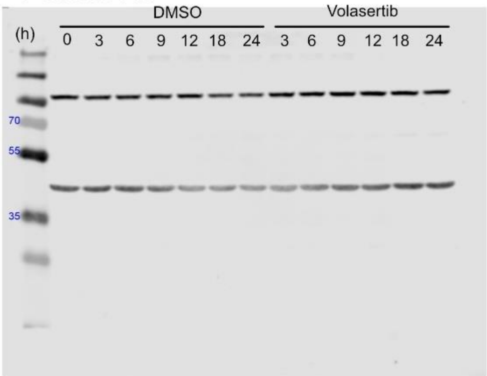

H1975: EGFR

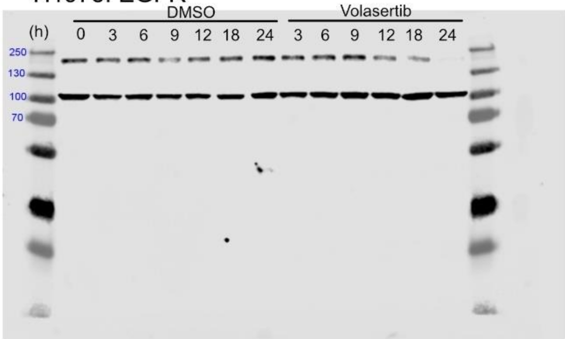

H1975: ACTIN

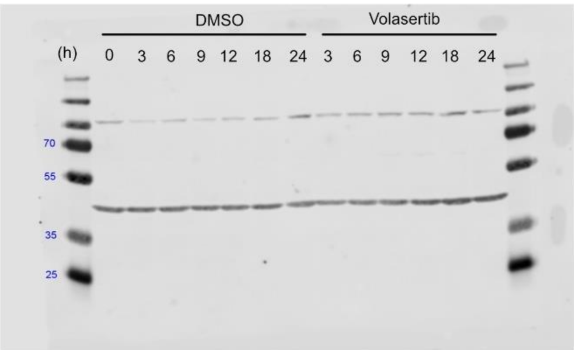

Uncropped Western blot from supplemental figure 3A

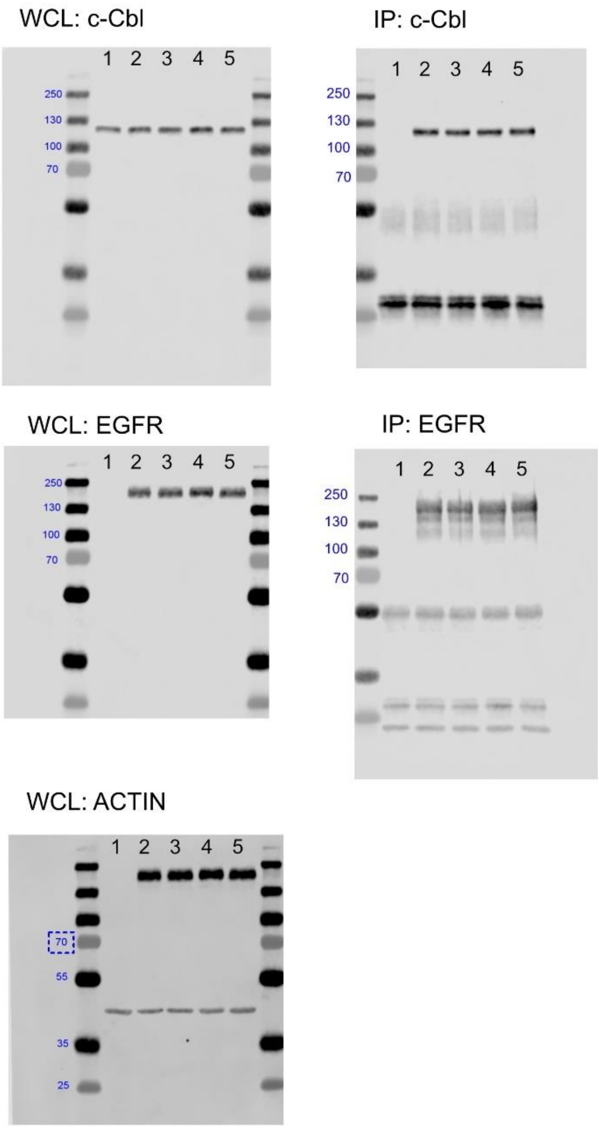

Lanes:

(1) *C-Cbl*

(2) *C-Cbl + EGFR-Δ746-750*

(3) *C-Cbl + EGFR-Δ746-750 + Volasertib 30min*

(4) *C-Cbl + EGFR-Δ746-750 + Volasertib 1h*

(5) *C-Cbl + EGFR-Δ746-750 + Volasertib 3h*

Uncropped Western blot from supplemental figure 3B

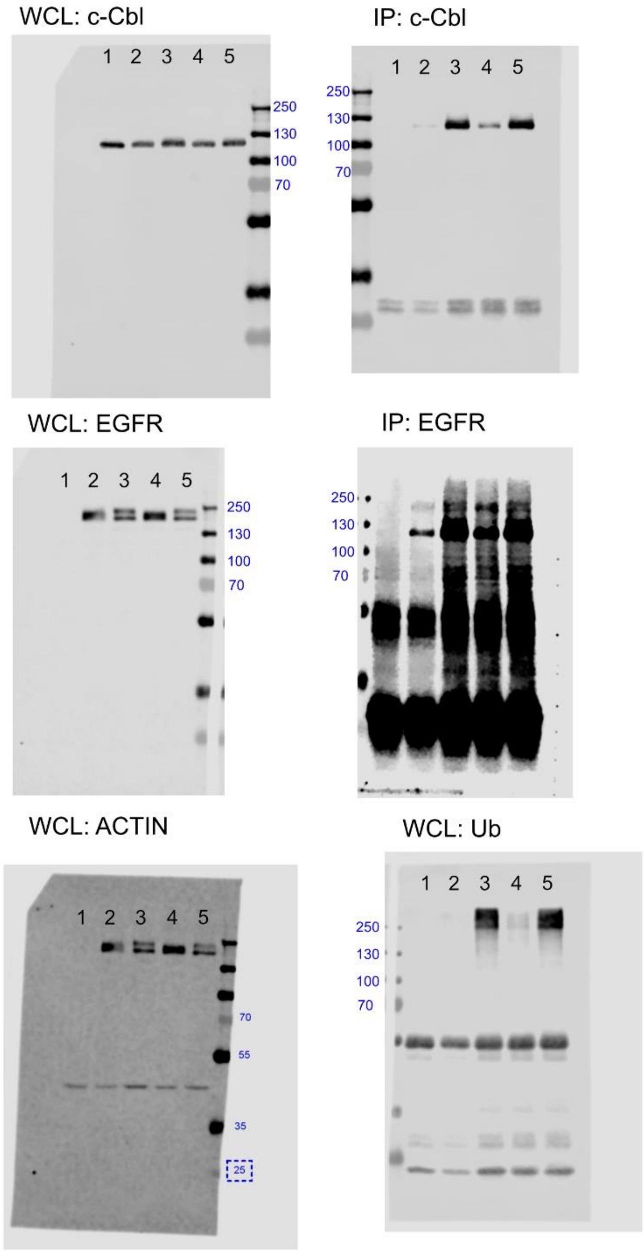

Lanes:

1. *C-Cbl*
2. *c-Cbl + EGFR-WT*
3. *c-Cbl + EGFR-WT + EGF*
4. *c-Cbl + EGFR-WT + Volasertib*
5. *c-Cbl + EGFR-WT + EGF + Volasertib*

Uncropped Western blot from supplemental figure 4A

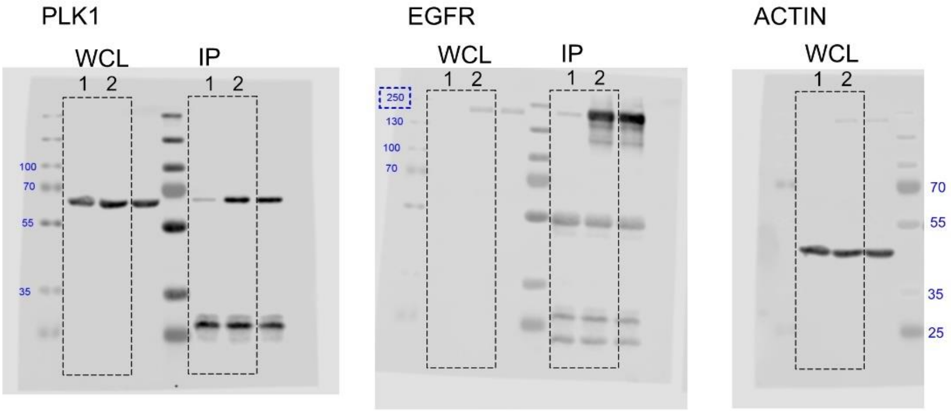

Lanes:

(1) PLK1-FLAG

(2) PLK1-FLAG + EGFR-Δ746-750

Uncropped Western blot from supplemental figure 4B

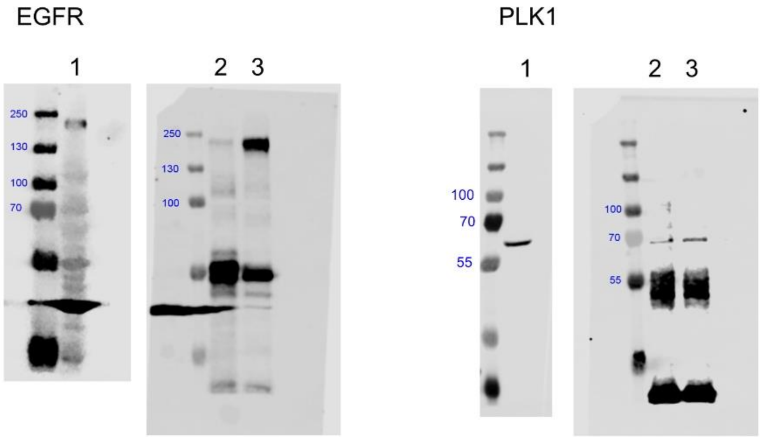

Lanes:

(1) WCL

(2) IP: IgG

(3) IP: EGFR
